# Supplementary material for: Sample observed effects: enumeration, randomization and generalization
Source: Sci Rep. 2025 Mar 11;15:8423. doi: 10.1038/s41598-024-80839-8 (PMC11897334; doi:10.1038/s41598-024-80839-8)
Supplement: Supplementary file 1 — Supplementary Information. [file 41598_2024_80839_MOESM1_ESM.pdf]

Sample Observed Effects: Enumeration, Randomization and Generalization (Appendix)

Andre F. Ribeiro‖

A Cause-Confounder Separation from Effect Observations

The possibility of observing the effect of sample factors across distinct orders is important to determine whether factors are confounders or credible causes, *Principle.1*. Consider a fixed permutation π_0 and another with a single transposition π_1 (i.e., with one inversion). According to *Principle.1*, each such operation can only reveal the appearance of one confounder. The expected variance in effect observations for a confounder b is, as consequence, proportional to the number of inversions between b and its root cause u, and inversely related to their correlation.

Along the same line, we said an effect observation is confounded when its effect can't be separated from the effect of all other factors in $Z = X \cup U$. Let x_0 be a population, we say a factor a is separated from b in the population x_0 when we have *observed* effects $\Delta y(x_0 - x)$ for all possible factor differences from x_0,

‖ribeiro@alum.mit.edu, Department of Applied Mathematics and Statistics, University of Sao Paulo.

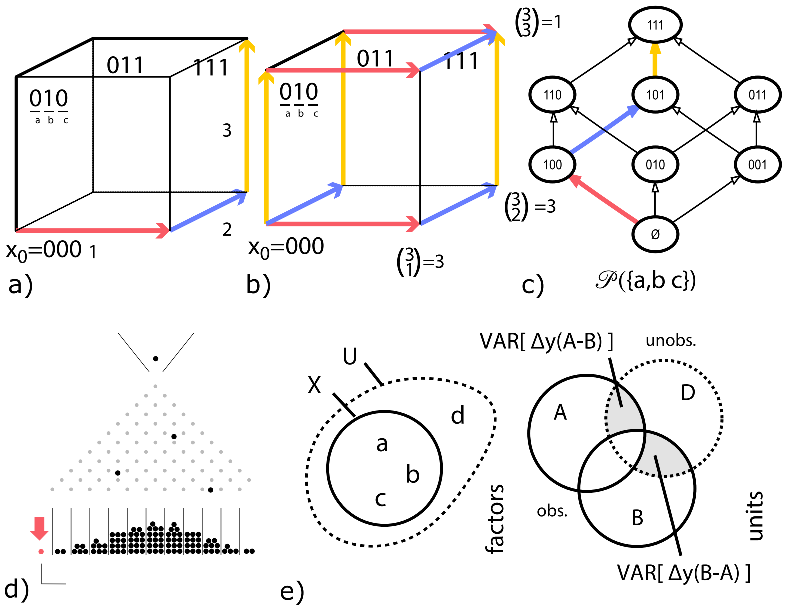

Supplementary Figure S1: **(a)** 3-dimensional cube and one $\{a, b, c\}$ path (colored), **(b)** 3 non-overlapping $\{a, b, c\}$ paths; **(c)** Hasse diagram for the power-set $\mathcal{P}(\{a, b, c\})$; **(d)** Galton-box and rare combination sampling; **(e)** example of sample ($m = 3$) with correlated and unobserved factors (left), and across-unit effect error decomposition (right).

$$\bigcup_{x \in \mathcal{P}(X)} \Delta y(x - x_0). \tag{23}$$

for all backgrounds. There are in a fully and partially samples, respectively, $\sum_{d=1}^{m} \binom{m}{d} = 2^m$ and $\sum_{d=1}^{m} \binom{m}{d} \times D_{q=m-d}$ unique partial permutations. The term D_m is the number of derangements, $D_m = m! \sum_{d=0}^{m} \frac{(-1)^d}{d!}$. The relationship indicates that, to form a partial permutation, we select d unique ('observed') factors to be organized in $\binom{m}{d}$ ways, each with $D_{q=m-d}$ possible disjoint orderings of the non-selected (non-'observed') factors. The relationship can be seen in the typical definition of factorials $m! = \sum_{t=0}^{m} \binom{m}{t} \times D_{m-t}$ [Ribeiro, 2022a].

There are two equivalent ways to visualize a single square. It is easy to visualize its resulting set of factor differences by placing the chosen reference population x_0 in one corner of a hypercube of dimension m, and its derangement in the opposite, Fig.S1(a). All hypercube vertices have m edges, each corresponding to a singleton difference and square letter. There are 2^m vertices, and m disjoint paths of size m starting at x_0 in a m-hypercube [Saad and Schultz, 1988], which correspond to square rows, Fig.S1(b). There are therefore m^2 cells in a square. The square can also be visualized with a Hasse diagram, Fig.S1(c). Eq.(23) enumerates all m-way factor differences from x_0 using the power-set of X. This makes x_0 the null element, $\emptyset$, of $\mathcal{P}(X)$, and leads to the familiar algebraic lattice for $\mathcal{P}(X)$ and the Hasse diagram. It is important to note that Eq.(23) makes the previous definitions for EV and CF independent on how variables and populations are coded (i.e., their individual binary values). No matter the chosen 'frame-of-reference' x_0 (small population, large population, etc.) all of its counterfactual observations must be observed.

Each *single* square thus contains all effect observations necessary to separate x_0, but only a single derangement and full permutation. These mutual effect observations can distinguish a's individual effect, $\Delta y(a|x_0)$, from the effect of every other factor combination, $x \in \mathcal{P}(X)$, without going beyond the observed data. This can be stated recursively. Let x_0 correspond to the element $\emptyset$ of the following enumeration. If we assume we can differentiate population $\{a\}$ from all others, $\mathcal{P}(X) - \{a\}$, then a single difference suffices

to differentiate $\emptyset$ and $\{a\}$. Such difference, $x_0 - x_i$, has specific properties,

$$\begin{aligned} &x_0 \cap x_i = x_0, \\ &x_i - x_0 = \{a\}, \\ &x_0 - x_i = \emptyset, \end{aligned} \tag{24}$$

where x_i is any population that fulfills these combinatorial properties with a fixed x_0. Reversely, we say that we need at least one such observation to separate confound x_0 and $x_0 - \{a\}$. The difference is a single *observation* of effects between x_0 and $x_0 - \{a\}$. If we next assume we can differentiate $\{a, b\}$ from all other sets, $\mathcal{P}(X) - \{a\} - \{a, b\}$, and $\emptyset$ from $\{a\}$, then all we need is a second difference with $x_0 \cap x_j = x_0 - \{a\}$, $x_j - x_0 = \{a, b\}$ and $x_0 - x_j = \emptyset$. This generates the difference

$$\begin{aligned} &x_i \cap x_j = x_i, \\ &x_j - x_i = \{b\}, \\ &x_i - x_j = \emptyset. \end{aligned} \tag{25}$$

The first difference was an observation of effect $\Delta y(a)$, and the second $\Delta y(b)$. The resulting sequence of differences is 'piecewise' and 'one-sided' - each difference with one commutation, $|x_i - x_j| = 1$, and another, $|x_j - x_i| = 0$. For a first-step difference, $x_i - x_0 = \{a\}$, there are $m-1$ other possible first steps, $\{b, c, ...\}$, and $m-1$ future steps until all factors are used, leading to m^2 unique differences. The set of all such differences thus leads to the complete set of differences from x_0, as illustrated by the Hasse diagram, each with its associated effect observation, Δy_{ij}. A CF-EV sequence provide us with means to do the same across all possible backgrounds, and describe when effects are expected to generalize across all possible conditions in $\{X, U\}$ system. In the second case, we cannot identify causes, only estimate the likelihood that in-sample factors are confounders.

B Separation and Effect Variance

Principle.1 starts with the definition of causes as perfect controls [Pearl, 2000]. It is easy to use the inclusion-exclusion principle in samples where all factors have the same frequency (square) to consider whether the residual variances of causes, confounders and spurious are expected to be different. Let a be an in-sample factor, $a \in X$, and d an out-of-sample cause, $d \in U$. If a is not correlated with d, then d affects all non-correlated factors equally, and this case is typically dealt with with large-sample assumptions. A more subtle case occurs when a is not a cause, but merely correlated with an out-of-sample cause d. In this case, the observed effect of a is not spurious, but can be nearly invariant, due to its association with d. We can imagine this relationship to be $\Delta y(a) = \rho \times \Delta y(d)$, with an unknown correlation ρ. When $\rho = 1$, a is either a perfect proxy or the cause itself, and there are no reasons to rule it out as such. When $\rho < 1$, there are contingencies in which the cause d is invariant, but the confounder a is not. We thus identify three cases for observed effect errors $\mathrm{Var}[\Delta y(a)]$: null for causes, constant (but not null) for confounders, and highly variable for spurious.

For the benefit of the reader, we outline the argument informally first. Fig.S1(e, right) shows a Venn diagram over sample units for the previous example (left), with one unobserved factor d, $U - X = \{d\}$. High-caps Latin letters indicate the population (set of units) that has the (low-caps) factor. Since squares contain effect observations for every combination of observed variables (for a given x_0), we can define effect estimates for each of these subpopulations and Venn partitions, except those overlapping D (i.e., $A \cap D$ and $B \cap D$). We do have any control over d, and, the factor can thus add extraneous variation to effect observations involving a and b. Such effect observations are subject to $p(d) \times \Delta y(d)$ expected biases. Consider effect observations $\Delta y(A - B)$ from x_0 to members of the $A - B$ population. We can write the variance in those effects as $\mathrm{Var}[\Delta y(A - B)] = \mathrm{Var}\{\Delta y[(A - B) - D] + \Delta y[(A - B) \cap D)]\}$. By definition, Eq.(3,4), $\Delta y[(A - B) - D]$ is constant, as we have discounted the effect of all observed causes and confounders, as well as the unobserved effect of D. Thus, effect variation from units in each of the $(A - B)$ and $(B - A)$ sections of the Venn diagram are due, exclusively, to external or unobserved variation, $\mathrm{Var}[\Delta y(A - B)] = \mathrm{Var}[\Delta y(D)] \times \rho_{ad}$. Effect variances across these sections define a distribution, whose positive support

indicates the expected effect of d on their respective effect observations, Fig.S1(e, top-right). Variance in these effect observations can thus identify confounders in X. Notice that this indicates that variance across square rows is associated with unobserved effect confounding, in the same way square diagonals are associated with observed confounding (*Sect.1.0.1 Squares and Observed Confounding*). Because the previous conditions are described by effect variances, $\mathrm{Var}[\Delta y(A{-}B)]$ and $\mathrm{Var}[\Delta y(B{-}A)]$, there is also a connection between this rationale and our EV definition, Eq.(4). We denote effect errors due to this catch-all unobserved variation $\mathrm{Var}_{(m-1)}$, and show it goes to zero in the case of full EV.

More generally, we can consider the variance of observed effects of a after discounting the variance of all other observed effects, Fig.S1(e) in samples where all factors are equally-represented. We write this variance as $\mathrm{Var}_{(m-1)}[\Delta y(a)]$, and formulate it with the inclusion-exclusion principle, and alternating sum,

$$\mathrm{Var}_{(m-1)}\Big[\,\Delta y\Big(\,a\mid\Pi(X-\{a\})\,\Big)\,\Big]$$
$$=\sum_{\substack{\pi\in\\ \Pi(X-\{a\})}}\left\{\sum_{\substack{x_i\in\\ \mathcal{P}_1^1(X-\{a\})}}\mathrm{Var}\Big[\Delta y\Big(a\,\Big|\,x|\pi\Big)\Big]-\sum_{\substack{x_i\in\\ \mathcal{P}_1^2(X-\{a\})}}\mathrm{Var}\Big[\Delta y\Big(a\,\Big|\,x|\pi\Big)\Big]+\ldots+(-1)^{m-1}\sum_{\substack{x_i\in\\ \mathcal{P}_1^{m-1}(X-\{a\})}}\mathrm{Var}\Big[\Delta y\Big(a\,\Big|\,x|\pi\Big)\Big]\right\}$$

The equation is a consequence of the equi-representation of combinations in the sample.

For one square and constant $\mathrm{Var}[\Delta y(a\,|\,x)]$ for any $x\in\mathcal{P}(X)$,

$$
\begin{aligned}
&\mathrm{Var}_{(m-1)}\Big[\,\Delta y\Big(\,a \mid \Pi(X-\{a\})\,\Big)\,\Big] \\
&= \binom{m-1}{1}\times\mathrm{Var}[\Delta y(a)] - \binom{m-1}{2}\times\mathrm{Var}[\Delta y(a)] + \ldots + (-1)^{m-1}\binom{m-1}{m-1}\times\mathrm{Var}[\Delta y(a)], \\
&= \Big[\sum_{i=1}^{m-1}(-1)^{i+1}\binom{m-1}{i}\Big]\times\mathrm{Var}[\Delta y(a)] = 0\times\mathrm{Var}[\Delta y(a)], \\
&= 0,
\end{aligned}
\tag{26}
$$

where the zero sum of the alternating binomial series, Eq.(26), is well known (*Appendix.E*). This is true either when $\mathrm{Var}[\Delta y(a)] = 0$ and a is a cause, Eq.(3,4), or, when $\mathrm{Var}[\Delta y(a)]$ is constant. This indicates that both causes and confounders have null residual variances in a sample with one square, in contrast to spurious out-of-sample factors.

For all squares,

$$
\begin{aligned}
&\mathrm{Var}_{(m-1)}\Big[\,\Delta y\Big(\,a \mid \Pi(X-\{a\})\,\Big)\,\Big] \\
&= 1!\binom{m-1}{1}\times\mathrm{Var}[\Delta y(a)] - 2!\binom{m-1}{2}\times\mathrm{Var}[\Delta y(a)] + \ldots + (-1)^{m-1}(m-1)!\binom{m-1}{m-1}\times\mathrm{Var}[\Delta y(a)], \\
&= \Big[(m-1)!\sum_{i=1}^{m-1}\frac{(-1)^i}{i!}\Big]\times\mathrm{Var}[\Delta y(a)], \\
&= D_{m-1}\times\mathrm{Var}[\Delta y(a)],
\end{aligned}
\tag{27}
$$

which is null only for causes, and grows linearly with the number of squares for confounders. This observation is only helpful when paired with the definition for causes (whether observed or not) in Eq.(4). Notice that Eq.(27) is associated with the alternating sum of effects in the square last column. It also implies that the **number of squares in samples indicate how well we can separate confounders and causes**. A sample with

few squares and derangements hold no such power. This has the practical consequence that correlated confounding can be detected by a test of linearity, a very well understood class of statistical tests. A summary statistic for this relationship is introduced in *Sect.G Bayesian Hierarchical Sample Errors Estimation*. In conclusion, this indicates that a single square can distinguish an in-sample confounder a from its root cause d by a $\Delta y(d)$ margin of $0.5 \times \rho_{ad}$, and multiple squares distinguish out-of-sample by $n_{sq} \times \rho_{ad}$. This can be visualized in a square by noting that confounders will have 0 or its root cause effect (adjusted by ρ_{ad}) depending whether it appears before or after its root cause, which happens in equal proportion. We used these observations, and *Principle.1*, to delineate limits to effect and importance attribution in partially-observed samples, across scenarios.

C Expected Number of Draws for All Binary Outcomes

This is an obvious result to many, and is included here for completeness. It gives the expected number of additional samples, after an arbitrary first, necessary to collect all binary outcomes under random sampling (i.e., obtain both heads, H, and tails, T, with a fair coin). Let x be the expected number of tosses to get HT after H was observed in the last flip. In that case, you can either observe T and be done, or observe H and be in the same situation, thus

$$x = \frac{1}{2} \cdot 1 + \frac{1}{2} \cdot (1 + x) \tag{28}$$

$$= 2. \tag{29}$$

D Number of Squares and Derangements

The recurrence defining derangements is $D_m = m\,(D_{m-1}) + (-1)^m$ (m>0, 1 otherwise). The total number of squares up to size m is

$$\sum_{d=1}^{m} \frac{D_m}{m} = \sum_{d=1}^{m} \left[\frac{(-1)^m}{m} + D_{m-1} \right], \tag{30}$$

$$= \ln(2) + \sum_{d=1}^{m} D_{m-1}, \tag{31}$$

$$= 0.69... + \sum_{d=1}^{m} D_{m-1}. \tag{32}$$

The total number of squares of m factors is thus less than a unit of the number of derangements of $m-1$. This is an error of around 0.002 for values as low as $m = 6$, and rapidly decreasing. From the recursion, the same is true for each m, ${}^{D_m}\!/_m - D_{m-1} = {}^{(-1)^m}\!/_m$.

E Alternating Binomial Coefficients Sum to Zero

This is a generally know result, $\sum_{i=0}^{n}(-1)^i\binom{n}{i} = 0$. For $n > 0$,

$$\sum_{i=0}^{n} (-1)^i \binom{n}{i} \tag{33}$$

$$= \binom{n}{0} + \sum_{i=1}^{n-1} (-1)^i \binom{n}{i} + (-1)^n \binom{n}{n}, \tag{34}$$

$$= \binom{n}{0} + \sum_{i=1}^{n-1} (-1)^i \left(\binom{n-1}{i-1} + \binom{n-1}{i} \right) + (-1)^n \binom{n}{n}, \tag{35}$$

$$= \binom{n}{0} - \sum_{i=1}^{n-1} \left((-1)^{i-1} \binom{n-1}{i-1} - (-1)^i \binom{n-1}{i} \right) + (-1)^n \binom{n}{n}, \tag{36}$$

$$= \binom{n}{0} - (-1)^{1-1} \binom{n-1}{1-1} + (-1)^{n-1} \binom{n-1}{n-1} + (-1)^n \binom{n}{n}, \tag{37}$$

$$= 1 - 1 + (-1)^{n-1} - (-1)^{n-1}, \tag{38}$$

$$= 0. \tag{39}$$

Eq.(35) is an application of Pascal's rule, Eq.(37) the sum of a telescoping series, and Eq.(38) of definitions of binomial coefficient with zero and self.

F Supervised Algorithms and Parameters

We perform hyperparameter searches over a variety of algorithms in order to find models that are not only accurate and generalizable, but also representative of contemporary and popular supervised techniques. In the tables below, we list typical hyperparameters that are randomly searched in optimizations.

We started with an algorithm stack with the following candidate methods [Tibshirani et al., 2001]: Cox Proportional Hazards (CoxPH), Deep Learning (Neural Networks), Distributed Random Forest (DRF), Generalized Linear Model (GLM), Maximum R Square Improvements (MAXR), Generalized Additive Models (GAM), ANOVA GLM, Gradient Boosting Machine (GBM), LASSO and Ridge regressions, Naïve Bayes Classifier, RuleFit, Support

Vector Machine (SVM), Extremely Randomized Forest (XRT) and XGBoost. Stacking (Super Learners) [Wolpert, 1992, Breiman, 1996] involves training a second-level metalearner to find the optimal combination of base learners. We also stacked all previous methods. We list optimized hyperparameters for methods that have featured at least once as leader (or were part of a leader Stacked Ensemble) in the article's tasks.

This table shows Generalized Linear Model (GLM) [Goldberger, 1962, Nelder and Wedderburn, 1972, Department et al.] parameter values that are searched over when performing grid search:

parameter	search
alpha	{0.0, 0.2, 0.4, 0.6, 0.8, 1.0}

This table shows XGBoost [Chen and Guestrin, 2016, Mitchell R, 2017] parameter values that are searched over when performing grid search:

parameter	search
booster	{gbtree, dart}
col sample rate	{0.7, 0.8, 0.9, 1.0}
max depth	{5, 10, 15, 20}
min rows	{0.01, 0.1, 1.0, 3.0, 5.0, 10.0, 15.0, 20.0}
ntrees	(binary search over [0,100000])
reg alpha	{0.001, 0.01, 0.1, 1, 10, 100}
sample rate	{0.6, 0.8, 1.0}

This table shows Gradient Boosting Machine (GBM) [Friedman et al., 2000, 2004, Elith et al., 2008] parameter values that are searched over when performing grid search (fixed values have a single value):

parameter	search
col sample rate	{0.4, 0.7, 1.0}
col sample rate per tree	{0.4, 0.7, 1.0}
learn rate	0.1
max depth	{3, 4, 5, 6, 7, 8, 9, 10, 11, 12, 13, 14, 15, 16, 17}
min rows	{1, 5, 10, 15, 30, 100}
min split improvement	{1e-4, 1e-5}
sample rate	{0.50, 0.60, 0.70, 0.80, 0.90, 1.00}
ntrees	(binary search over [0,100000])
sample rate	{0.50, 0.60, 0.70, 0.80, 0.90, 1.00}

This table shows Deep Learning [Baldi and Hornik, 1989, Kuhn et al., 2005] parameter values that are searched over when performing grid search:

parameter	search
activation	Rectifier with Dropout
epochs	10000 (true value found by early stopping)
epsilon	{1e-6, 1e-7, 1e-8, 1e-9}
hidden grid	{{20}, {50}, {100},
	{20, 20}, {50, 50}, {100, 100},
	{20, 20, 20}, {50, 50, 50}, {100, 100, 100}}
hidden dropout ratios	{ {0.1}, {0.2}, {0.3}, {0.4}, {0.5},
	{0.1, 0.1}, {0.2, 0.2}, {0.3, 0.3}, {0.4, 0.4}, {0.5, 0.5},
	{0.1, 0.1, 0.1}, {0.2, 0.2, 0.2}, {0.3, 0.3, 0.3}, {0.4, 0.4, 0.4}, {0.5, 0.5, 0.[illegible]
input dropout ratio	{0.0, 0.05, 0.1, 0.15, 0.2}
rho	{0.9, 0.95, 0.99}

G Bayesian Hierarchical Sample Errors Estimation

We use a hierarchical Bayesian approach to estimate errors due to unobserved confounding in Eq.(9). This is done only to establish further connections to

other work and to generate Fig.7(p,q). Eq.(9) suggests decomposing errors for each square cell (k, d) as $\varepsilon_{a,d} = \varepsilon_a + \varepsilon_d$, where a is the cell's allocated variable and ε_d is the cell 'gap' error - introduced by the distinct number of confounders across distinct ordinal sample conditions (square's columns). In a EV-increasing sample the effect of every variable a is observed at every 'position' (i.e., across all gaps),

$$\varepsilon_a \perp \varepsilon_d,$$

when there are no omitted variables in samples.

Often this is not the case, the previous errors are not independent. In samples with the proposed conditions, however, Eq.(9) suggests that is possible to assume a common and additive component ε_d across all effect observations. EV-increasing and CF non-increasing sampling sequences are key to this solution as they lets sample factors be subject to common and known amounts of out of sample variation, and the external variation be more easily parsed out.

More specifically, a square is a fully nested design. In nested designs, observations are not assumed i.i.d., but have correlations. With a nested approach, the variation introduced in each hierarchy level is assessed relative to the level below it. A nested Analysis of Variance (also called a hierarchical ANOVA) is an ANOVA extension that imposes a separate correlation structure within each nest. The resulting hierarchical Bayesian model is

$$\begin{aligned} \Delta^d y(a) &\sim \alpha_a^d + \epsilon_{col}^d + \epsilon_{sq}, \\ \epsilon_{sq}, \epsilon_{col}^d &\sim \mathcal{N}(0, \sigma_{sq}^2), \mathcal{N}(0, \sigma_{col}^{2,d}), \\ \sigma_{sq}, \sigma_{col}^d &\sim Cauchy(0, 25) \\ \alpha_a^d, \alpha_b^d, ... &\sim \mathcal{N}(0, 10^5), \\ d &\sim \mathcal{U}[\{i, i(j), i(k), i(j), i(k), i(jk), ...\}], \end{aligned} \tag{40}$$

where $\Delta^d y(a)$ is the observed effect of factor a (resp. b, c, ...), α_a its estimated effect, n the number of samples drawn, and the ϵ terms are error

components. The first error component, ϵ^d_{col}, is the error associated with the d-th square column, and ϵ_{sq} is the unexplained, across-columns, component. Both are assumed to be normally distributed with zero mean and constant standard deviation in this illustrative parametric model. The assumptions of Normality are unproblematic as is well-known that U-Statistics are asymptotically Normal [Hoeffding, 1948]. Superscripts are sample unit iterators. Sometimes they are used in nested ANOVA to indicate the nestedness among subpopulations. The index $i(j)$ indicates that population j is nested in a superpopulation ij. Here, they are also used as remainder that units are sampled uniformly from the set of unique nestings. Each statistic with a superscript is consequently a per-column statistics. Variables without indices indicate across-squares statistics. The index d is sampled uniformly from the discrete set of indices $d \in \{i, i(j), i(k), i(j), i(k), i(jk), ...\}$. We can think of this Bayesian approach as first randomly choosing a square column, updating statistics for all singleton and column's effects, and repeating. After a large number of repetitions, singleton effects correspond to effect estimates under all distinct nestings, and estimated over square diagonals. This model is estimated with variational techniques [Carpenter et al., 2017].

H Sample Sizes

Let us start with the problem of collecting the first square (or any one square), then consider the problem of collecting many squares. **We expect EV to increase with the latter**. The first corresponds to the problem of collecting all differences (combinatorial combinations) for a fixed unit x_0. In a square, there are 2^m unique combinations, and 2^m draws are necessary to collect them without replacement. When sampling individual factors randomly - i.e., $p(a) = 0.5$ for all $a \in X$ - the expected number of draws with replacement required for a full square will be higher than without replacement, as for each factor, we can obtain a value that is the same, or different, from ones in the past. The process described in *Sect.6 Sample Sizes* for sampling combinations can be visualized with a Galton board. As illustrated in Fig.S1(d), the board consists of rows of pegs that create multiple paths for marbles. Marbles are dropped at the top and can take either the rightward (+1) or leftward (−1) path with equal probability when they encounter a peg. At the bottom,

the marbles accumulate in a set of bins, which reproduces asymptotically Pascal's triangle. The rare event in the Galton board corresponds to a ball hitting its extremal slots, and the probability of sampling these is at the order of 2^{m-1} times more difficult than a ball hitting a central slot. That is, combinations at the upper and lower tails of the board are the most difficult to sample. Since we need to obtain exactly all combinations, the worst-case sample requirement corresponds to the cost of finding these combinations. With a simple application of the pigeon-hole principle, once we obtain these combinations, we have likely already collected all previous combinations, and a full square.

Let us formalize further, and generalize the previous discussion to the non-random case. Let $\mathbf{M}$ be the probability of a factor's least likely value (its 'minimal'), $\mathbf{M} = \min\{\mathbf{p}(a), \mathbf{p}(\bar{a})\}$, with cumulative distribution function Φ. Let also M_t denote this probability after t factor draws. The exact distribution for this value is

$$\begin{aligned} \Pr(M_t \leq M) &= \Pr(\mathbf{M}_1 \leq M, \ldots, \mathbf{M}_t \leq M), \\ &= \Pr(\mathbf{X}_1 \leq M) \cdots \Pr(\mathbf{M}_t \leq M), \\ &= (\Phi(M))^t, \end{aligned}$$

where M is an arbitrary probability value, $0 \leq M \leq 1$. The quantity $\Phi(M)^t$ represents the probability that all draws taken have a value less than or equal to M (i.e., the definition of a minimum).

The associated indicator function $I_t = I(M_t > M)$ is a Bernoulli process with a success probability $p_M = 1 - (\Phi(M))^t$ that depends on the true probability, M, of the minimal. (i.e., the last ordinal $X_{(m-1)}$) Consider then the number of factor draws necessary to obtain a minimum when its true value is M. The number of minimal draws within t trials follows a binomial distribution, and the number of trials until a minimal draw follows a geometric distribution with expected value of the order of its reciprocal, $O(1/p_M)$. That is, let N_M be the count of trials until the factor minimum is obtained. Let $p_{\bar{M}}$ be the probability that we have not sampled the minimum $p_{\bar{M}} = 1 - p_M$. According to the Law of Iterated Expectations, N_M can be defined inductively from the outcome of the first draw. If we do not get a minimum then, we

are up one count and the experiment repeats; otherwise, the experiment ends and the count is 1. So, the expected number of draws is defined recursively as

$$\begin{aligned}\mathbb{E}[N_M] &= p_{\bar{M}} \times (1 + \mathbb{E}[N_M]) + (1 - p_{\bar{M}}) \times 1,\\ &= \frac{1}{1 - p_{\bar{M}}} = \frac{1}{p_M},\\ &= \lambda.\end{aligned}$$

An equiprobable sample has $n_a = n_b = ... = n_{[m]}$. These correspond to fully balanced designs in the Experimental literature [Montgomery, 2001], to the operation of the Galton board, and the gap-probability case $s = 0$ in Eq.(**??**). In this equiprobable case, the number of expected draws to obtain both values, $\{-1, +1\}$, is $\lambda = 2$ (*Appendix.C*). We need these many draws for the minimal factor, repeated across all the 2^{m-1} combinations of other factors, to guarantee we sample the combinations at the board tails. Therefore, $\tilde{n} = 2^{m-1} \times 2 = 2^m$. For non-equiprobable samples, the number of draws necessary for each factor correspond to the number of draws required for its rarest factor, λ_{min}. In this case, $\lambda_{min} > (0.5)^{-1}$ and the number of samples is thus increasing. Regardless, we need λ_{min} draws to guarantee we obtain both values, $\{-1, +1\}$ in the worst case (i.e., for the rarest factor). This number of draws is repeated for each of the 2^{m-1} combinations not including the minimal. At the end, we have collected both values for all factors, across all their combinations, and thus a full, but arbitrary, square.

The minimum λ_{min} can be defined in two ways, as an exact value or an approximation to across-combinations minimal factor frequency. We considered effect estimation where factors can have higher-order interactions in effect. We can assume that there are no interactions in factor *frequencies* - e.g., $p(a) \approx p(a \,|\, b)$ - and choose λ_{min} as the frequency of the sample rarest factor. Because we enumerate sample combinations, we can also choose the λ_{min} that corresponds to the rarest factor and combination pair. We find that, with the former, sample sizes in Eq.(21) provide simple and useful order-of-magnitude sample size guides for effect estimation (*Sect.7 Experiments*).

H.1 Multiple Squares

Let us say we now have a first square, sampled as described in the previous section. To generate a next square for the same population x_0 (combination) we need to re-sample all other combinations, while keeping x_0 fixed. We can write the number of observations necessary to accomplish this as fixing one combination (x_0) and repeating the process in the last section for all remaining combinations,

$$\left(2^{m-1} - \frac{1}{2^{m-1}}\right).$$

If we repeat this many times, to generate many squares, we have

$$\left(2^{m-1} - \frac{1}{2^{m-1}}\right)^t \xrightarrow{t\to\infty} \phi, \qquad (D_m \gg m) \tag{41}$$

where Eq.(41) is a known expression for the golden ratio ϕ[1]. When ${}^{D_m}/_{m}$ is large (in relation to m), this generates many new squares. It then leads to the asymptotic limit in Eq.(21)(right) for many squares. It suggests minimal sizes for samples with high EV for a single population x_0. This simple result can also be stated purely combinatorially [Ribeiro, 2022a] (as the diagonal of number in Pascal's triangles follow a golden ration), or from repeating arithmetic series [Ribeiro, 2022b]. Sample size asymptotic requirements are therefore simple, and reflect directly the previous combinatorial structures. The first number in Eq.(21) corresponds to the cost of sampling all combinations (differences) from a reference, Eq.(23), and the latter many such differences with the same reference, Eq.(3). Each of these is multiplied by λ_{min} to guarantee that all individual factors are collected in an unbalanced sample, following the previous arguments from Extreme Value theory.

[1] $\left(m - \frac{1}{m}\right)^t \to \phi$ for a constant m and increasing t, see, for example, [Gazale, 1999].

I Enumeration

To enumerate squares, we first enumerate all observed permutations in a sample then assemble them into squares. The first is simple: the set of all pairs of units with singleton differences, $a \in X$, are placed in the first column of a square-like matrix. Then all pairs with difference $b \in X$ and overlap a are placed in the second column (and in the row containing a). If there are r unique differences b (with overlap a) at this stage, the procedure adds $r-1$ new rows to the matrix. The process is repeated for all subsequent singleton pairwise differences and antecedent overlaps. After m repetitions, each matrix row contains a sample permutation.

To assemble these permutations into squares, we first code each (partial) permutation with a Lehmer code. Richard Korf proposed a way of encoding permutations in linear time when proposing a Rubik's cube solver [Korf and Schultze, 2005]. It converts the Lehmer code into a base-10 number. Indices for partial permutations can be obtained in the same way, but with one difference. For a full permutation, each digit in the Lehmer code has a base of $(m-1-i)!$, where i is the digit position. For a partial permutation, the base of each digit is D^{d-1-i}_{m-1-i}, where d is the number of items fixed. The procedure generates a unique index for all partial permutations and derangements. We also create an inverted index with (row number, permutation code). From each permutation it is then easy to find all its unique rotations. To enumerate squares, it suffices to select all permutations with full squares (m rotations) and follow their unique rotation order. Due to the indexing of partial permutations, this also allows for the enumeration of incomplete and partial squares (as used in *Sect.7 Experiments*). Enumerated this way, squares enumerate populations (i.e., not its members). Given the observed sample, population members are indistinguishable but may have different y_i. When enumerating square population members, we pick them without replacement. When $m>10$, this process is repeated hierarchically, hashing permutations based on their overlaps to all previous permutations. Permutations are, this way, represented by sets of $\log_{10}(m)$ indices, and consequently, sets of permutations with increasing fixed-points.

References

K. Aas, M. Jullum, and A. Løland. Explaining individual predictions when features are dependent: More accurate approximations to shapley values. *Artificial Intelligence*, 298:103502, 2021. doi: https://doi.org/10.1016/j.artint.2021.103502. URL `https://www.sciencedirect.com/science/article/pii/S0004370221000539`.

A. Abadie. Using synthetic controls: Feasibility, data requirements, and methodological aspects. *Journal of Economic Literature*, 59(2):391–425, June 2021. doi: 10.1257/jel.20191450. URL `https://www.aeaweb.org/articles?id=10.1257/jel.20191450`.

A. Abadie and G. W. Imbens. Large sample properties of matching estimators for average treatment effects. *Econometrica*, 74(1):235–267, 2006. doi: 10.1111/j.1468-0262.2006.00655.x.

S. Amari, N. Murata, K.-R. Müller, M. Finke, and H. Yang. Statistical theory of overtraining: Is cross-validation asymptotically effective? In *Proceedings of the 8th International Conference on Neural Information Processing Systems*, NIPS'95, pages 176–182, Cambridge, MA, USA, 1995. MIT Press.

Y. Bahri, E. Dyer, J. Kaplan, J. Lee, and U. Sharma. Explaining neural scaling laws, 2021.

P. Baldi and K. Hornik. Neural networks and principal component analysis: Learning from examples without local minima. *Neural networks*, 2(1):53–58, 1989. doi: 10.1016/0893-6080(89)90014-2.

D. Bayer and P. Diaconis. Trailing the dovetail shuffle to its lair. *The Annals of Applied Probability*, 2(2):294–313, 1992.

C. Bénesse, F. Gamboa, J.-M. Loubes, and T. Boissin. Fairness seen as global sensitivity analysis. *Machine Learning*, 113(5):3205–3232, 2024. doi: 10.1007/s10994-022-06202-y. URL `https://doi.org/10.1007/s10994-022-06202-y`.

A. Bietti and J. Mairal. Group invariance, stability to deformations, and complexity of deep convolutional representations. *J. Mach. Learn. Res.*, 20 (1):876–924, jan 2019. ISSN 1532-4435.

L. Breiman. Stacked regressions. *Machine learning*, 24(1):49, 1996. doi: 10.1023/A:1018046112532.

P. Buehlmann. Invariance, causality and robustness. *Statistical science*, 35 (3):404–426, 2020. doi: 10.1214/19-STS721.

J. Buhler and R. Graham. *Juggling patterns, passing, and posets*, volume Mathematical adventures for students and amateurs /. Mathematical Association of America, [Washington, DC] :, c2004.

N. Burkart and M. F. Huber. A survey on the explainability of supervised machine learning. *J. Artif. Int. Res.*, 70:245–317, may 2021. ISSN 1076-9757. doi: 10.1613/jair.1.12228. URL `https://doi.org/10.1613/jair.1.12228`.

C. Bycroft, C. Freeman, D. Petkova, G. Band, L. T. Elliott, K. Sharp, A. Motyer, D. Vukcevic, O. Delaneau, J. O'Connell, A. Cortes, S. Welsh, A. Young, M. Effingham, G. McVean, S. Leslie, N. Allen, P. Donnelly, and J. Marchini. The uk biobank resource with deep phenotyping and genomic data. *Nature*, 562(7726):203–209, 2018. doi: 10.1038/s41586-018-0579-z. URL `https://doi.org/10.1038/s41586-018-0579-z`.

S. Calonico, M. D. Cattaneo, M. H. Farrell, and R. Titiunik. Regression Discontinuity Designs Using Covariates. *The Review of Economics and Statistics*, 101(3):442–451, 07 2019. ISSN 0034-6535. doi: 10.1162/rest_a_00760. URL `https://doi.org/10.1162/rest\_a\_00760`.

B. Carpenter, A. Gelman, M. D. Hoffman, D. Lee, B. Goodrich, M. Betancourt, M. Brubaker, J. Guo, P. Li, and A. Riddell. Stan: A probabilistic programming language. *Journal of Statistical Software, Articles*, 76(1):1–32, 2017. ISSN 1548-7660. doi: 10.18637/jss.v076.i01. URL `https://www.jstatsoft.org/v076/i01`.

C. S. D. CDC-Dataset-2021. https://data.cdc.gov/case-surveillance/covid-19-case-surveillance-public-use-data-with-ge/n8mc-b4w4.

A. Chatton, F. Le Borgne, C. Leyrat, F. Gillaizeau, C. Rousseau, L. Barbin, D. Laplaud, M. Leger, B. Giraudeau, and Y. Foucher. G-computation, propensity score-based methods, and targeted maximum likelihood estimator for causal inference with different covariates sets: a comparative

simulation study. *Nature Scientific reports*, 10(1):9219–9219, 2020. doi: 10.1038/s41598-020-65917-x.

T. Chen and C. Guestrin. Xgboost: A scalable tree boosting system. In *Proceedings of the 22nd ACM SIGKDD International Conference on Knowledge Discovery and Data Mining*, KDD '16, pages 785–794, New York, NY, USA, 2016. Association for Computing Machinery. ISBN 9781450342322. doi: 10.1145/2939672.2939785. URL `https://doi.org/10.1145/2939672.2939785`.

Y. S. Chen, P. P. Chong, and M. Y. Tong. Mathematical and computer modelling of the pareto principle. *Mathematical and Computer Modelling*, 19(9):61–80, 1994. doi: https://doi.org/10.1016/0895-7177(94)90041-8. URL `https://www.sciencedirect.com/science/article/pii/0895717794900418`.

T. S. Cohen and M. Welling. Group equivariant convolutional networks. In *Proceedings of the 33rd International Conference on International Conference on Machine Learning - Volume 48*, ICML'16, pages 2990–2999. JMLR.org, 2016.

T. H. Cormen. *Introduction to algorithms.* MIT Press : McGraw-Hill, Cambridge, Mass.; Boston, 2001. ISBN 0262032937.

J. Correa and E. Bareinboim. General transportability of soft interventions: Completeness results. In H. Larochelle, M. Ranzato, R. Hadsell, M. Balcan, and H. Lin, editors, *Advances in Neural Information Processing Systems*, volume 33, pages 10902–10912. Curran Associates, Inc., 2020. URL `https://proceedings.neurips.cc/paper_files/paper/2020/file/7b497aa1b2a83ec63d1777a88676b0c2-Paper.pdf`.

D. R. Cox. Randomization in the design of experiments. *International Statistical Review*, 77(3):415–429, 2024/03/03 2009. doi: https://doi.org/10.1111/j.1751-5823.2009.00084.x. URL `https://doi.org/10.1111/j.1751-5823.2009.00084.x`.

E. Y. Cramer, V. K. Lopez, J. Niemi, G. E. George, J. C. Cegan, I. D. Dettwiller, W. P. England, M. W. Farthing, R. H. Hunter, B. Lafferty, I. Linkov, M. L. Mayo, M. D. Parno, M. A. Rowland, B. D. Trump, L. Wang, L. Gao, Z. Gu, M. Kim, Y. Wang, J. W. Walker, R. B. Slayton,

M. Johansson, and M. Biggerstaff. Evaluation of individual and ensemble probabilistic forecasts of covid-19 mortality in the us, 2021.

P. de Boer and J. F. D. Rodrigues. Decomposition analysis: when to use which method? *Economic systems research*, 32(1):1–28, 2020. doi: 10.1080/09535314.2019.1652571.

B. Department, N. E. Breslow, C. Nazionale, C. Convegni, and S. Agostino. Generalized linear models: Checking assumptions and strengthening conclusions.

P. Diaconis. The cutoff phenomenon in finite markov chains. *Proceedings of the National Academy of Sciences*, 93(4):1659–1664, 2024/02/26 1996. doi: 10.1073/pnas.93.4.1659. URL https://doi.org/10.1073/pnas.93.4.1659.

P. Diaconis and J. Fulman. *The Mathematics of Shuffling Cards.* American Mathematical Society, 2023. ISBN 9781470463038. URL https://books.google.co.uk/books?id=dB2_EAAAQBAJ.

P. Diaconis, R. L. Graham, and W. M. Kantor. The mathematics of perfect shuffles. *Advances in Applied Mathematics*, 4(2):175–196, 1983. doi: https://doi.org/10.1016/0196-8858(83)90009-X. URL https://www.sciencedirect.com/science/article/pii/019688588390009X.

J. Elith, J. R. Leathwick, and T. Hastie. A working guide to boosted regression trees. *Journal of Animal Ecology*, 77(4):802–813, 2021/09/16 2008. doi: https://doi.org/10.1111/j.1365-2656.2008.01390.x. URL https://doi.org/10.1111/j.1365-2656.2008.01390.x.

U. N. Emeruwa, S. Ona, J. L. Shaman, A. Turitz, J. D. Wright, C. Gyamfi-Bannerman, and A. Melamed. Associations Between Built Environment, Neighborhood Socioeconomic Status, and SARS-CoV-2 Infection Among Pregnant Women in New York City. *JAMA*, 324(4):390–392, 07 2020. ISSN 0098-7484. doi: 10.1001/jama.2020.11370. URL https://doi.org/10.1001/jama.2020.11370.

A. F. Ribeiro, F. Neffke, and R. Hausmann. What can the millions of random treatments in nonexperimental data reveal about causes? *Springer Nature Computer Science*, 3(6):421, 2022. doi: 10.1007/s42979-022-01319-2. URL https://doi.org/10.1007/s42979-022-01319-2.

J. Friedman, T. Hastie, and R. Tibshirani. Special invited paper. additive logistic regression: A statistical view of boosting. *The Annals of statistics*, 28(2):337–374, 2000.

J. Friedman, T. Hastie, S. Rosset, R. Tibshirani, and J. Zhu. [consistency in boosting]: Discussion. *The Annals of statistics*, 32(1):102–107, 2004.

J. Fulman. The combinatorics of biased riffle shuffles. *Combinatorica*, 18 (2):173–184, 1998. doi: 10.1007/PL00009814. URL `https://doi.org/10.1007/PL00009814`.

M. J. Gazale. *Gnomon: from pharaohs to fractals*. Princeton University Press, Princeton, N.J, 1999. ISBN 0691005141; 9780691005140.

A. S. Goldberger. Best linear unbiased prediction in the generalized linear regression model. *Journal of the American Statistical Association*, 57(298): 369–375, 1962. doi: 10.1080/01621459.1962.10480665.

J. Grimmer, D. Knox, and B. M. Stewart. Na\"ive regression requires weaker assumptions than factor models to adjust for multiple cause confounding. 2020.

A. Gut. *An Intermediate Course in Probability*. Springer Publishing Company, Incorporated, 2nd edition, 2009. ISBN 1441901612.

I. Guyon. A scaling law for the validation-set training-set size ratio. In *AT and T Bell Laboratories*, 1997.

D. J. Hand and R. J. Till. A simple generalisation of the area under the roc curve for multiple class classification problems. *Machine Learning*, 45(2): 171–186, 2001. doi: 10.1023/A:1010920819831. URL `https://doi.org/10.1023/A:1010920819831`.

D. Hanson, K. Seyffarth, and J. H. Weston. Matchings, derangements, rencontres. *Mathematics Magazine*, 56(4):224–229, 1983. doi: 10.2307/2689812.

W. Hoeffding. A class of statistics with asymptotically normal distribution. *The Annals of mathematical statistics*, 19(3):293–325, 1948. doi: 10.1214/aoms/1177730196.

J. Jin, N. Agarwala, P. Kundu, B. Harvey, Y. Zhang, E. Wallace, and N. Chatterjee. Individual and community-level risk for covid-19 mortality in the united states. *Nature Medicine*, 27(2):264–269, 2021. doi: 10.1038/s41591-020-01191-8. URL `https://doi.org/10.1038/s41591-020-01191-8`.

M. Kearns. A bound on the error of cross validation using the approximation and estimation rates, with consequences for the training-test split. In *Proceedings of the 8th International Conference on Neural Information Processing Systems*, NIPS'95, pages 183–189, Cambridge, MA, USA, 1995. MIT Press.

A. P. Keil, J. K. Edwards, D. B. Richardson, A. I. Naimi, and S. R. Cole. The parametric g-formula for time-to-event data: Intuition and a worked example. *Epidemiology*, 25(6), 2014. URL `https://journals.lww.com/epidem/fulltext/2014/11000/the_parametric_g_formula_for_time_to_event_data_.16.aspx`.

O. Kempthorne and T. E. Doerfler. The behaviour of some significance tests under experimental randomization. *Biometrika*, 56(2):231–248, 08 1969. ISSN 0006-3444. doi: 10.1093/biomet/56.2.231. URL `https://doi.org/10.1093/biomet/56.2.231`.

D. E. Knuth. *The art of computer programming, volume 2 (3rd ed.): seminumerical algorithms*. Addison-Wesley Longman Publishing Co., Inc., USA, 1997. ISBN 0201896842.

R. E. Korf and P. Schultze. Large-scale parallel breadth-first search. In *Proceedings of the 20th National Conference on Artificial Intelligence - Volume 3*, AAAI'05, pages 1380–1385. AAAI Press, 2005. ISBN 157735236x.

R. R. Kuhn, P. P. Sollich, and A. C. C. A. C. C. . Coolen. *Theory of neural information processing systems*. Oxford University Press, Oxford, 2005. ISBN 0198530234; 9780198530237; 0198530242; 9780198530244.

Y. A. LeCun, L. Bottou, G. B. Orr, and K.-R. Müller. *Efficient BackProp*, pages 9–48. Springer Berlin Heidelberg, Berlin, Heidelberg. ISBN 0302-9743. doi: 10.1007/978-3-642-35289-8{_}3.

A. J. Lee. *U-statistics : theory and practice*. M. Dekker, New York, 1990. ISBN 0824782534.

Y. Li, M. A. Horowitz, J. Liu, A. Chew, H. Lan, Q. Liu, D. Sha, and C. Yang. Individual-level fatality prediction of covid-19 patients using ai methods. *Frontiers in Public Health*, 8:566, 2020. ISSN 2296-2565. doi: 10.3389/fpubh.2020.587937. URL `https://www.frontiersin.org/article/10.3389/fpubh.2020.587937`.

S. M. Lundberg and S.-I. Lee. A unified approach to interpreting model predictions. In *Proceedings of the 31st International Conference on Neural Information Processing Systems*, NIPS'17, pages 4768–4777, Red Hook, NY, USA, 2017. Curran Associates Inc. ISBN 9781510860964.

S. M. Lundberg, G. G. Erion, and S.-I. Lee. Consistent individualized feature attribution for tree ensembles, 2019.

S. Magliacane, T. van Ommen, T. Claassen, S. Bongers, P. Versteeg, and J. M. Mooij. Domain adaptation by using causal inference to predict invariant conditional distributions. 2017.

F. E. Mitchell R. Accelerating the xgboost algorithm using gpu computing. In *PeerJ Computer Science*, volume 3:e127, 2017.

D. C. Montgomery. *Design and analysis of experiments.* John Wiley, New York, 2001. ISBN 0471316490; 9780471316497.

S. L. Morgan and C. Winship. *Counterfactuals and Causal Inference: Methods and Principles for Social Research.* Cambridge University Press, Cambridge, 2007. ISBN 0521671930; 9780521856157; 9780521671934; 0521856159. doi: 10.1017/CBO9780511804564.

J. A. Nelder and R. W. M. Wedderburn. Generalized linear models. *Journal of the Royal Statistical Society, Series A, General*, 135:370–384, 1972.

J. Pearl. *Causality : models, reasoning, and inference.* Cambridge, U.K. ; New York, 2000. ISBN 0521773628. Includes bibliographical references (p. 359-373) and indexes.; ID: http://id.lib.harvard.edu/aleph/008372583/catalog.

J. Pearl and E. Bareinboim. Transportability of causal and statistical relations: A formal approach. In *2011 IEEE 11th International Conference on Data Mining Workshops*, pages 540–547, 2011. doi: 10.1109/ICDMW.2011.169.

J. Pearl and E. Bareinboim. External validity: From do-calculus to transportability across populations. *Statistical Science*, 29(4):579–595, 11 2014. doi: 10.1214/14-STS486. URL https://doi.org/10.1214/14-STS486.

J. Peters, P. Bühlmann, and N. Meinshausen. Causal inference by using invariant prediction: identification and confidence intervals. *Journal of the Royal Statistical Society. Series B, Statistical methodology*, 78(5):947–1012, 2016. doi: 10.1111/rssb.12167.

B. Rader, S. V. Scarpino, A. Nande, A. L. Hill, B. Adlam, R. C. Reiner, D. M. Pigott, B. Gutierrez, A. E. Zarebski, M. Shrestha, J. S. Brownstein, M. C. Castro, C. Dye, H. Tian, O. G. Pybus, and M. U. G. Kraemer. Crowding and the shape of covid-19 epidemics. *Nature Medicine*, 26(12):1829–1834, 2020. doi: 10.1038/s41591-020-1104-0. URL https://doi.org/10.1038/s41591-020-1104-0.

A. Rényi. On the theory of order statistics. *Acta Mathematica Academiae Scientiarum Hungarica*, 4(3):191–231, 1953. doi: 10.1007/BF02127580. URL https://doi.org/10.1007/BF02127580.

A. F. Ribeiro. Spatiocausal patterns of sample growth, 2022a. URL https://arxiv.org/abs/2202.13961.

A. F. Ribeiro. Population structure and effect generalization, 2022b. URL https://arxiv.org/abs/2209.13560.

P. R. Rosenbaum and D. B. Rubin. The central role of the propensity score in observational studies for causal effects. *Biometrika*, 70(1):41–55, 1983. doi: 10.1093/biomet/70.1.41.

D. B. Rubin. Causal inference using potential outcomes: Design, modeling, decisions. *Journal of the American Statistical Association*, 100(469):322–331, 2005. doi: 10.1198/016214504000001880.

Y. Saad and M. H. Schultz. Topological properties of hypercubes. *IEEE Transactions on Computers*, 37(7):867–872, 1988. doi: 10.1109/12.2234.

B. Scholkopf, F. Locatello, S. Bauer, N. R. Ke, N. Kalchbrenner, A. Goyal, and Y. Bengio. Toward causal representation learning. *Proceedings of the IEEE*, 109(5):612–634, 2021. doi: 10.1109/JPROC.2021.3058954.

U. Shalit, F. D. Johansson, and D. Sontag. Estimating individual treatment effect: generalization bounds and algorithms. In D. Precup and Y. W. Teh, editors, *Proceedings of the 34th International Conference on Machine Learning*, volume 70 of *Proceedings of Machine Learning Research*, pages 3076–3085. PMLR, 06–11 Aug 2017. URL http://proceedings.mlr.press/v70/shalit17a.html.

I. M. Sobol. Global sensitivity indices for nonlinear mathematical models and their monte carlo estimates. *Mathematics and Computers in Simulation*, 55(1):271–280, 2001. doi: https://doi.org/10.1016/S0378-4754(00)00270-6. URL https://www.sciencedirect.com/science/article/pii/S0378475400002706.

R. Tibshirani, J. H. J. H. . Friedman, and T. Hastie. *The elements of statistical learning : data mining, inference, and prediction.* Springer, New York, 2001. ISBN 0387952845.

U. B. D. UKB-Showcase-2021. https://biobank.ndph.ox.ac.uk/showcase/.

M. van der Laan and S. Rose. *Targeted Learning: Causal Inference for Observational and Experimental Data*. Springer Series in Statistics. Springer New York, 2011. ISBN 9781441997821. URL https://books.google.co.uk/books?id=RGnSX5aCAgQC.

R. Verity, L. C. Okell, and Dorigatti. Estimates of the severity of coronavirus disease 2019: a model-based analysis (vol 20, pg 669, 2020). *The Lancet infectious diseases*, 20(6):E116–E116, 2020. doi: 10.1016/S1473-3099(20)30309-1.

Y. Wang and D. M. Blei. The blessings of multiple causes. *Journal of the American Statistical Association*, 114(528):1574–1596, 2020. doi: 10.1080/01621459.2019.1686987.

D. H. Wolpert. Stacked generalization. *Neural networks*, 5(2):241–259, 1992. doi: 10.1016/S0893-6080(05)80023-1.

H. Yamato and Y. Maesono. Invariant u-statistics. *Communications in statistics. Theory and methods*, 15(11):3253–3263, 1986. doi: 10.1080/03610928608829307.

Y. Zhang and Q. Zhao. What is a randomization test? *Journal of the American Statistical Association*, 118(544):2928–2942, 10 2023. doi: 10.1080/01621459.2023.2199814. URL https://doi.org/10.1080/01621459.2023.2199814.
